# Supplementary material for: Content-rich biological network constructed by mining PubMed abstracts
Source: BMC Bioinformatics. 2004 Oct 8;5:147. doi: 10.1186/1471-2105-5-147 (PMC528731; doi:10.1186/1471-2105-5-147)
Supplement: Additional File 2 — The original results of the above study (non-essential files are deleted to keep the file size under the limit set by BMC bioinformatics). [file 1471-2105-5-147-S2.bz2 › chilibotAdditionalFile2/dip50/377ID9927421E9790/html/NGFR_NTRK1.html]

 


 **NGFR** and **NTRK1** 
  
Found 541 abstracts in PubMed, retrieved 50.  
 

 What does Google say? 
 PDF only 
| .edu only 

---

**Interactive relationship** (e.g. stimulation, inhibition, etc)

**Neutral relationship**- The signal transducing systems that mediate the diverse biological functions of the neurotrophins are initiated by their interactions with two categories of cell surface receptors, the Trk  [ **NTRK1** ]  family of tyrosine kinases and the p75 neurotrophin receptor p75NTR  [ **NGFR** ] .  Ref: 12169297 Prog Neurobiol, 2002
- The adjacent nerves expressed the high affinity NGF receptor  [ **NGFR** ]  trk A  [ **NTRK1** ] .  Ref: 12115873 J Pathol, 2002
- Adrenergic mouse pheochromocytoma MPC cells from heterozygous neurofibromatosis knockout mice show little or no expression of the NGF receptor  [ **NGFR** ]  trk A  [ **NTRK1** ]  and do not undergo neuronal differentiation in response to NGF.  Ref: 12438155 Ann N Y Acad Sci, 2002
- Double staining revealed that almost all trkA  [ **NTRK1** ]  immunoreactive ON cells also contained p75NTR  [ **NGFR** ]  immunoreactivity.  Ref: 12175342 Neuropathol Appl Neurobiol, 2002
- Their actions on neural tissue are thought to be mediated by interaction with high affinity trk  [ **NTRK1** ]  family members or low affinity p75NTR  [ **NGFR** ]  cell surface receptors.  Ref: 12141445 Int J Dev Biol, 2002
- Overall, our results indicate an essential role for p75NTR  [ **NGFR** ]  in supporting NGF triggered TrkA  [ **NTRK1** ]  signaling pathways mediating neuronal survival in hippocampal neurons.  Ref: 12453482 Neuroscience, 2002
- trk  [ **NTRK1** ]  neu consists of the extracellular domain of the trkA  [ **NTRK1** ]  nerve growth factor NGF receptor  [ **NGFR** ]  fused to the transmembrane and cytoplasmic domains of c erbB2, allowing NGF induced c erbB2 homodimer signaling.  Ref: 12181354 Mol Biol Cell, 2002
- In addition, a new interaction in the TrkA  [ **NTRK1** ]  p75NTR  [ **NGFR** ]  heteromeric receptor signal transduction network was revealed, namely that NGF induced activation of the MAPK pathway appears to inhibit the parallel NGF induced Akt pathway.  Ref: 12702729 J Biol Chem< MedlineTA>J Biol Chem, 2002
- Although neurotrophins did not regulate p75NTR  [ **NGFR** ]  processing, the alpha and gamma secretase mediated cleavage of p75 is modulated by receptor tyrosine kinases Trks TrkA  [ **NTRK1** ]  and TrkB but not TrkC.  Ref: 12843241 J Neurosci, 2003
- A chimera of the nerve growth factor NGF receptor  [ **NGFR** ] , TrkA  [ **NTRK1** ] , and green fluorescent protein GFP was engineered by expressing GFP in phase with the carboxyl terminus of TrkA  [ **NTRK1** ] .  Ref: 12438306 J Biol Chem, 2003
- Previous studies from this laboratory and others have suggested the evidences that acidic glycosphingolipid, ganglioside GM1 GM1, is an endogenous regulator of high affinity nerve growth factor receptor  [ **NTRK1** ]   [ **NGFR** ] , Trk  [ **NTRK1** ] , which is an essential factor for the normal development and differentiation of neuronal cells by forming a complex with Trk  [ **NTRK1** ] .  Ref: 12374216 Neurochem Res, 2002
- In addition, the effect of albumin seemed to depend on the activity of the NGF receptor  [ **NGFR** ] , TrkA  [ **NTRK1** ] , suggesting that the glutamate synthesized and released due to the presence of albumin promotes neuronal survival through the activity of TrkA  [ **NTRK1** ] .  Ref: 12065647 J Neurochem, 2002
- First, p75NTR  [ **NGFR** ]  is a Trk  [ **NTRK1** ]  co receptor that can enhance or suppress neurotrophin mediated Trk  [ **NTRK1** ]  receptor activity.  Ref: 12169297 Prog Neurobiol, 2002
- RESULTS We found that RA increased the expression of both trkA  [ **NTRK1** ]  and p75 NGF receptors in NCI N 592 and GLC8 cell lines and prevented the loss of both NGF production and NGF receptor  [ **NGFR** ]  expression occurring when NGF treatment was discontinued.  Ref: 12213675 Eur J Endocrinol, 2002
- To address this issue, various combinations of transfected PC12 cells expressing a PDGFR TrkA  [ **NTRK1** ]  chimera and the p75NTR  [ **NGFR** ]  selective NGF mutant D9 13 NGF were utilized to selectively stimulate TrkA  [ **NTRK1** ]  or p75NTR  [ **NGFR** ]  signaling, respectively.  Ref: 12702729 J Biol Chem< MedlineTA>J Biol Chem, 2002
- On the one hand, p75NTR  [ **NGFR** ]  provides a positive modulatory influence on nerve growth factor NGF signaling through the high affinity neurotrophin receptor TrkA  [ **NTRK1** ] , and hence increases NGF survival signaling.  Ref: 12453482 Neuroscience, 2002
- While activation of TrkA  [ **NTRK1** ]  signaling is sufficient for eliciting neurite outgrowth in PC12 cells, signaling through p75NTR  [ **NGFR** ]  plays a modulatory role, especially in the increased formation of fine, synaptic bouton like structures, in which both TrkA  [ **NTRK1** ]  and p75NTR  [ **NGFR** ]  appear to co localize.  Ref: 12702729 J Biol Chem< MedlineTA>J Biol Chem, 2002

**Non-interactive relationship** (e.g. studied together, co-existance, homology, etc.)

- Here we show that survival of sympathetic neurons supported by NGF on distal axons requires the kinase activity of the NGF receptor  [ **NGFR** ] , TrkA  [ **NTRK1** ] , in both distal axons and cell bodies.  Ref: 12848932 Neuron, 2003
- Pharmacological and dominant negative inhibition and kinase assays indicate that Bgamma promotes neuritogenesis by stimulating the MAP kinase cascade downstream of the TrkA  [ **NTRK1** ]  NGF receptor  [ **NGFR** ]  but upstream or at the level of the B Raf kinase.  Ref: 12191994 J Biol Chem, 2002
- SHB mediates certain responses in platelet derived growth factor PDGF receptor, fibroblast growth factor FGF receptor, neural growth factor NGF receptor  [ **NGFR** ]  TRKA  [ **NTRK1** ] , T cell receptor, interleukin 2 IL 2 receptor and focal adhesion kinase FAK signaling.  Ref: 12776987 Curr Mol Med, 2003
- Expression and Activation of the Nerve Growth Factor Receptor  [ **NGFR** ]  TrkA  [ **NTRK1** ]  in Serous Ovarian Carcinoma.  Ref: 12796393 Clin Cancer Res< MedlineTA>Clin Cancer Res, 2003
- A long standing question in neurotrophin signal transduction is whether heteromeric TrkA  [ **NTRK1** ]  p75NTR  [ **NGFR** ]  complexes possess signaling capabilities that are significantly different from homo oligomeric TrkA  [ **NTRK1** ]  or p75NTR  [ **NGFR** ]  alone.  Ref: 12702729 J Biol Chem< MedlineTA>J Biol Chem, 2003
- We have used immunofluorescence techniques with a panel of antibodies against known neurotrophin receptors trk A  [ **NTRK1** ] , trk  [ **NTRK1** ]  B, trk  [ **NTRK1** ]  C, p75NTR  [ **NGFR** ]  to map the locations of these receptors in the developing neuromuscular system of mice from our neurotrophin 3 NT 3 knockout colony.  Ref: 12141445 Int J Dev Biol, 2002
- However, p75NTR  [ **NGFR** ]  may also signal independently of TrkA  [ **NTRK1** ] , causing cell death or cell survival, depending on the cell type and stage of development.  Ref: 12453482 Neuroscience, 2002
- The immunohistochemical localization of nerve growth factor receptor  [ **NGFR** ]   **NGFR**  high affinity  **NGFR**  trk A  [ **NTRK1** ]  and low affinity  **NGFR**  LNGFR was investigated in 23 neuroblastoma group tumors, 18 pheochromocytomas, 2 mixed neuroendocrine neural tumors, and 16 retinoblastomas.  Ref: 12114799 Endocr Pathol< MedlineTA>Endocr Pathol, 2002
- However, NGF rescue from growth factor withdrawal requires NGF signalling through the high affinity tyrosine kinase receptor  [ **NTRK1** ]  TrkA  [ **NTRK1** ] , while NGF rescue from TNFalpha promoted cell death requires NGF signalling through the low affinity p75NTR  [ **NGFR** ]  receptor.  Ref: 12882321 Aging Cell, 2003
- Endogenous NGF was responsible for the basal levels of trkA  [ **NTRK1** ]   **NGFR**  and ERK1 2 phosphorylation observed in untreated HUVEC cultures.  Ref: 12154004 FASEB J, 2002
- The contrary response of different cells to neurotrophins in terms of cell cycle regulation derives in part from the fact that these factors use two different receptor types to transmit their signals members of the Trk  [ **NTRK1** ]  family and the p75 neurotrophin receptor p75NTR  [ **NGFR** ] .  Ref: 12371150 Histol Histopathol, 2002
- Astrocytes have been implicated in axon guidance and synaptic regeneration in the retina and these processes involve activation of the high affinity nerve growth factor receptor  [ **NTRK1** ]   [ **NGFR** ] , known as the tyrosine kinase A receptor.  Ref: 12536040 Neurosci Lett, 2003
- Here we present evidence that the nerve growth factor receptor  [ **NGFR** ]  TrkA  [ **NTRK1** ]  may also promote phosphorylation of APP.  Ref: 12150951 Biochem Biophys Res Commun, 2002
- Mouse PTPRO mRNA was observed in neuron populations expressing TrkA  [ **NTRK1** ] , the high affinity nerve growth factor receptor  [ **NTRK1** ]   [ **NGFR** ] , or TrkC, the neurotrophin 3 receptor, and immunoreactive mPTPRO and TrkC colocalized in large dorsal root ganglia proprioceptive neurons.  Ref: 12532410 J Comp Neurol, 2003
- By contrast, no trkA  [ **NTRK1** ]  or p75NTR  [ **NGFR** ]  immunoreactivity was detected in nasopharyngeal carcinoma cells from five patients.  Ref: 12175342 Neuropathol Appl Neurobiol, 2002
- Increased expression of nerve growth factor NGF and high affinity NGF receptor  [ **NGFR** ]  p140 TrkA  [ **NTRK1** ]  in human osteoarthritic chondrocytes.  Ref: 12468822 Rheumatology Oxford , 2002
- RESULTS These results revealed a low amount of NGF in the plasma of old subjects associated with a reduced expression of mRNA for NGF receptor  [ **NGFR** ]  trkA  [ **NTRK1** ]  in peripheral lymphocytes.  Ref: 12679610 Gerontology< MedlineTA>Gerontology, 2002
- In the older GK rats, both mRNA and protein expression of nerve growth factor in the sciatic nerve were significantly reduced, and  **NGFR**  TrkA  [ **NTRK1** ]  and NGFRp75 protein expression was reduced in dorsal root ganglia.  Ref: 12469361 Diabetes Metab Res Rev< MedlineTA>Diabetes Metab Res Rev, 2002
- The NGF receptor  [ **NGFR** ]  TrkA  [ **NTRK1** ]  was found on all examined tumor cell lines and is not restricted to cells belonging to the nervous system.  Ref: 12148587 J Exp Clin Cancer Res, 2002
- NGF treatment caused a rapid phosphorylation of trkA  [ **NTRK1** ]   **NGFR**  in HUVECs, determining a parallel increase of phosphorylated ERK1 2.  Ref: 12154004 FASEB J, 2002
- Although neither TrkA  [ **NTRK1** ]  nor p75NTR  [ **NGFR** ]  was detectable in either HCC or normal hepatic cells, TrkA  [ **NTRK1** ]  was shown in the walls of tumor associated arteries that contain abundant nerve fibers.  Ref: 12055603 Gastroenterology, 2002
- Rat visual cortical neurones express TrkA  [ **NTRK1** ]  NGF receptor  [ **NGFR** ] .  Ref: 12151805 Neuroreport, 2002
- RT PCR analysis and immunological methods demonstrate that human umbilical vein endothelial cells HUVECs express the NGF receptors trkA  [ **NTRK1** ]   **NGFR**  and p75NTR  [ **NGFR** ] .  Ref: 12154004 FASEB J, 2002
- In mouse hippocampal cells lacking p75NTR  [ **NGFR** ]  p75NTR  [ **NGFR** ]  activation of TrkA  [ **NTRK1** ]  by NGF was not detectable.  Ref: 12453482 Neuroscience, 2002
- METHODS Retroviral vectors were constructed to express the high affinity NGF receptor  [ **NGFR** ]  TrkA  [ **NTRK1** ]  and low affinity NGF receptor  [ **NGFR** ]  p75 NTR.  Ref: 12877805 Chin Med J (Engl), 2003
- Thus, a chimeric receptor was created that possessed the extracellular and transmembrane domains of the nerve growth factor NGF receptor  [ **NGFR** ]  TrkA  [ **NTRK1** ]  and the cytoplasmic domain of PDGFRbeta TTbeta.  Ref: 12794304 J Mol Neurosci, 2003
- After 2 weeks of treatment of PLA cells with isobutylmethylxanthine, indomethacin, and insulin, about 20 to 25 percent of the cells differentiated into cells with typical neural morphologic characteristics, accompanied by increased expression of NSE, vimentin, and the nerve growth factor receptor  [ **NGFR** ]  trk A  [ **NTRK1** ] .  Ref: 12711954 Plast Reconstr Surg, 2003
- Tissue Localization of Nerve Growth Factor Receptors trk A  [ **NTRK1** ]  and Low Affinity Nerve Growth Factor Receptor  [ **NGFR** ]  in Neuroblastoma, Pheochromocytoma, and Retinoblastoma.  Ref: 12114799 Endocr Pathol< MedlineTA>Endocr Pathol, 2003
- Accordingly, NGF induced a significant increase in HUVEC proliferation that was abolished by the trkA  [ **NTRK1** ]   **NGFR**  inhibitor K252a.  Ref: 12154004 FASEB J, 2002
- The neurotrophins are able to bind to two different receptors all bind to a common receptor p75NTR  [ **NGFR** ] , and each also binds to one of a family of Trk  [ **NTRK1** ]  receptors.  Ref: 12787319 Neuropathol Appl Neurobiol, 2003
- PURPOSE The purpose is to analyze the possible correlation between expression and activation of the high affinity nerve growth factor NGF receptor  [ **NGFR** ]  TrkA  [ **NTRK1** ] , cell cycle protein expression, and disease outcome in serous ovarian carcinoma.  Ref: 12796393 Clin Cancer Res< MedlineTA>Clin Cancer Res, 2003
- In the present study, the effect of signaling generated from the Erb B2 homodimer as a result of transcription of the MUC1 gene was investigated in human mammary epithelial cell lines MTSV1 7 and Hb2 stably transfected with a pBAT trk  [ **NTRK1** ]  neu construct in which the extracellular domain of Erb B2 was replaced with the corresponding domain from the nerve growth factor NGF receptor  [ **NGFR** ] .  Ref: 12798775 Cell Biol Int, 2003
- P75NTR  [ **NGFR** ]  signaling independent of TrkA  [ **NTRK1** ] , such as increased neutral sphingomyelinase NSMase activity causing enhanced levels of ceramide, were not detected after exposure of hippocampal neurons to NGF.  Ref: 12453482 Neuroscience, 2002
- METHODS Expression of NGF, TrkA  [ **NTRK1** ] , and p75NTR  [ **NGFR** ]  was investigated in HCCs developing and regenerating livers, and primary hepatocyte cultures in B6C3F 1 mice by reverse transcription polymerase chain reaction, Northern blotting, and or immunohistochemistry.  Ref: 12055603 Gastroenterology, 2002
- Detection of NGF receptors TrkA  [ **NTRK1** ]  and p75NTR  [ **NGFR** ]  in human tumor cell lines and effect of NGF on the growth characteristic of the UT 7 EPO cell line.  Ref: 12148587 J Exp Clin Cancer Res, 2002
- Insulin receptor related receptor expression is tightly coupled to the nerve growth factor receptor  [ **NGFR** ] , TrkA  [ **NTRK1** ] , throughout development.  Ref: 12111796 J Neurosci Res, 2002
- Evidence for a role of the nerve growth factor receptor  [ **NGFR** ]  TrkA  [ **NTRK1** ]  in tyrosine phosphorylation and processing of beta APP.  Ref: 12150951 Biochem Biophys Res Commun, 2002
- Individual and combined effects of TrkA  [ **NTRK1** ]  and p75NTR  [ **NGFR** ]  nerve growth factor NGF receptors A role for the high affinity receptor site.  Ref: 12702729 J Biol Chem< MedlineTA>J Biol Chem, 2002
- 5 immunohistochemistry study revealed that the expression of nerve growth factor receptor  [ **NGFR** ]  trk A  [ **NTRK1** ]  was induced by acupuncture after cerebral ischemia, which may play a role in antagonizing apoptosis.  Ref: 12532826 Sheng Li Ke Xue Jin Zhan, 1999
- Quantitative in situ hybridization was used to detect changes in neuronal PACAP mRNA expression and to correlate this expression with neuronal mRNA expression of the NGF receptor  [ **NGFR** ]  tyrosine kinase trk A  [ **NTRK1** ] .  Ref: 12890505 Neuroscience, 2003
- The receptors for nerve growth factor NGF TrkA  [ **NTRK1** ]  and p75NTR  [ **NGFR** ]  were detected at the mRNA and the protein level in various human tumor cell lines.  Ref: 12148587 J Exp Clin Cancer Res, 2002
- OBJECTIVE We aimed to investigate the expression of nerve growth factor NGF and high affinity NGF receptor  [ **NGFR** ]  p140 TrkA  [ **NTRK1** ]  on chondrocytes from human healthy and osteoarthritic cartilage.  Ref: 12468822 Rheumatology Oxford , 2002
- Pathways responsible for transducing the sensitisation of TRPV1 by TrkA  [ **NTRK1** ] , the NGF receptor  [ **NGFR** ] , were characterised by observing the effects of inhibitors of key members of NGF activated second messenger signalling cascades.  Ref: 12815188 J Physiol< MedlineTA>J Physiol, 2002
- While the Trk  [ **NTRK1** ]  receptors are responsible for most of the survival and growth properties of the neurotrophins, the actions of p75NTR  [ **NGFR** ]  fall into two categories.  Ref: 12169297 Prog Neurobiol, 2002
- This response reached maximal levels as early as 5 min after the initiation of the coincubation and was completely blocked by anti NGF Ab or by an inhibitor for a tyrosine kinase of the trkA  [ **NTRK1** ]  NGF receptor  [ **NGFR** ] .  Ref: 12055260 J Immunol, 2002
- Ten cases of ON and five cases of nasopharyngeal carcinoma were examined for expression of trkA  [ **NTRK1** ]  and p75 neurotrophin receptor p75NTR  [ **NGFR** ]  using immunohistochemistry and double labelling fluorescence.  Ref: 12175342 Neuropathol Appl Neurobiol, 2002
- We found that all ON tissues from 10 cases expressed both trkA  [ **NTRK1** ]  and p75NTR  [ **NGFR** ]  at different levels.  Ref: 12175342 Neuropathol Appl Neurobiol, 2002
- These results suggest that nerve growth factor may play a role in the generation of ON and staining of trkA  [ **NTRK1** ]  and p75NTR  [ **NGFR** ]  may assist in the diagnosis of ON.  Ref: 12175342 Neuropathol Appl Neurobiol, 2002
- There was no detectable expression of high affinity tyrosine kinase receptor  [ **NTRK1** ]  trkA  [ **NTRK1** ]   **NGFR** .  Ref: 12580757 J Mol Endocrinol, 2003
- Biochemical and molecular analyses of the expression, fate, and kinetics of membrane trafficking of the nerve growth factor NGF receptor  [ **NGFR** ]  TrkA  [ **NTRK1** ]  were performed in PC12 cells.  Ref: 12055187 J Biol Chem, 2002
- PURPOSE To determine the presence of nerve growth factor NGF, NGF mRNA, and NGF receptor  [ **NGFR** ]  TrkA  [ **NTRK1** ]  in rabbit ocular tissues, and whether changes occur in NGF and NGF mRNA levels after experimental iridectomy.  Ref: 12091435 Invest Ophthalmol Vis Sci, 2002
- CONCLUSIONS This is the first study showing that human chondrocytes synthesize NFG beta and express on their surface the high affinity  **NGFR**  p140 TrkA  [ **NTRK1** ] .  Ref: 12468822 Rheumatology Oxford , 2002
